# Omega-3 Fatty Acids and Inflammation: Novel Interactions Reveal a New Step in Neutrophil Recruitment

**DOI:** 10.1371/journal.pbio.1000177

**Published:** 2009-08-25

**Authors:** Samantha P. Tull, Clara M. Yates, Benjamin H. Maskrey, Valerie B. O'Donnell, Jackie Madden, Robert F. Grimble, Philip C. Calder, Gerard B. Nash, G. Ed. Rainger

**Affiliations:** 1 Centre for Cardiovascular Sciences, School of Clinical and Experimental Medicine, The Medical School, The University of Birmingham, Birmingham, United Kingdom; 2 Department of Medical Biochemistry and Immunology, School of Medicine, Cardiff University, Cardiff, United Kingdom; 3 Institute of Human Nutrition, School of Medicine, University of Southampton, Southampton, United Kingdom; NIH/NIAID, United States of America

## Abstract

Inflammation is a physiological response to tissue trauma or infection, but leukocytes, which are the effector cells of the inflammatory process, have powerful tissue remodelling capabilities. Thus, to ensure their precise localisation, passage of leukocytes from the blood into inflamed tissue is tightly regulated. Recruitment of blood borne neutrophils to the tissue stroma occurs during early inflammation. In this process, peptide agonists of the chemokine family are assumed to provide a chemotactic stimulus capable of supporting the migration of neutrophils across vascular endothelial cells, through the basement membrane of the vessel wall, and out into the tissue stroma. Here, we show that, although an initial chemokine stimulus is essential for the recruitment of flowing neutrophils by endothelial cells stimulated with the inflammatory cytokine tumour necrosis factor-α, transit of the endothelial monolayer is regulated by an additional and downstream stimulus. This signal is supplied by the metabolism of the omega-6-polyunsaturated fatty acid (n-6-PUFA), arachidonic acid, into the eicosanoid prostaglandin-D_2_ (PGD_2_) by cyclooxygenase (COX) enzymes. This new step in the neutrophil recruitment process was revealed when the dietary n-3-PUFA, eicosapentaenoic acid (EPA), was utilised as an alternative substrate for COX enzymes, leading to the generation of PGD_3_. This alternative series eicosanoid inhibited the migration of neutrophils across endothelial cells by antagonising the PGD_2_ receptor. Here, we describe a new step in the neutrophil recruitment process that relies upon a lipid-mediated signal to regulate the migration of neutrophils across endothelial cells. PGD_2_ signalling is subordinate to the chemokine-mediated activation of neutrophils, but without the sequential delivery of this signal, neutrophils fail to penetrate the endothelial cell monolayer. Importantly, the ability of the dietary n-3-PUFA, EPA, to inhibit this process not only revealed an unsuspected level of regulation in the migration of inflammatory leukocytes, it also contributes to our understanding of the interactions of this bioactive lipid with the inflammatory system. Moreover, it indicates the potential for novel therapeutics that target the inflammatory system with greater affinity and/or specificity than supplementing the diet with n-3-PUFAs.

## Abstract 2

While investigating new mechanisms by which the dietary omega-3 fatty acids regulate inflammation, the authors have identified a new step in the regulation of neutrophil migration across vascular endothelial cells.

## Author Summary

Inflammation is a physiological response to tissue trauma or infection. Neutrophils, which circulate in the blood stream, are the first inflammatory cells to be recruited to a site of tissue inflammation. In response to recruitment signals provided by chemotactic peptides called chemokines, neutrophils traverse the endothelial cell lining of blood vessels. This process involves a multistep cascade of neutrophil adhesion and activation events on the endothelial barrier. While investigating the anti-inflammatory functions of the omega-3 fatty acid , EPA, which is found, for instance, in dietary fish oil, we identified an additional unexpected lipid-derived signal that is essential for neutrophil migration across the endothelium. Our experiments show that a chemokine delivered the first signal needed to bind neutrophils firmly to the endothelial surface. However, in order to traverse the endothelium, a subsequent signal delivered by prostaglandin-D_2_ (PGD_2_), a lipid derived from the omega-6 fatty acid arachidonic acid, was essential. When EPA, was introduced into the experiment, it was used to form PGD_3_. This alternative lipid blocked interactions between PGD_2_ and its receptor on neutrophils, preventing the process of migration across the endothelial barrier. Thus, we reveal a new step in the recruitment of neutrophils during inflammation, and a novel anti-inflammatory mechanism of action of dietary EPA.

## Introduction

**Figure 1:**
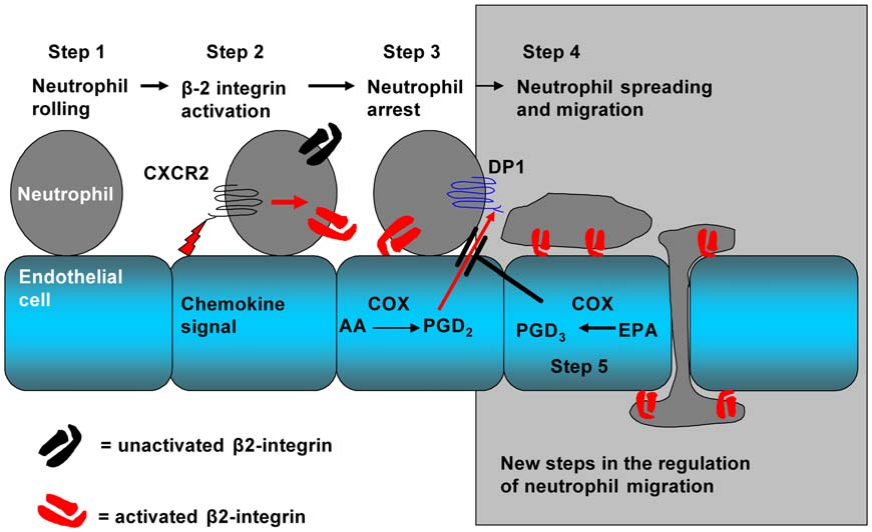
Steps in the process of neutrophil recruitment. Step 1: Neutrophils are captured from flow and tether and roll on tumour necrosis factor-α (TNF)-stimulated endothelial cells. Step 2: Neutrophils are then activated by the action of CXC-chemokines on the chemokine receptor, CXCR2, a process resulting in the activation of neutrophil β2-integrins. Step 3: β2-integrins engage counter receptors on the endothelial cell surface, and the neutrophil becomes stationary. Step 4: Prostaglandin-D_2_ (PGD_2_), generated by the action of cyclooxygenase enzymes on the n-6-PUFA arachidonic acid (AA), binds the PGD_2_ receptor, DP-1. DP-1 generates signals that stabilise neutrophil adhesion, induce neutrophil shape change, and support the process of transmigration across the endothelial cell monolayer. Step 5: If the endothelial cells have been supplemented with the n-3-PUFA, eicosapentaenoic acid (EPA), the alternative series prostanoid, PGD_3_, is generated, and this antagonises PGD_2_-mediated neutrophil responses.

**Figure 2:**
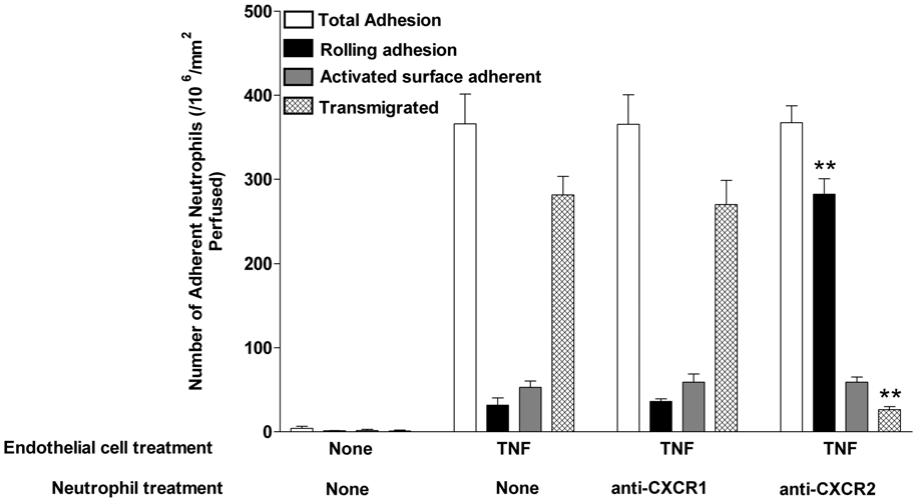
Chemokine signalling through CXCR2 is essential for neutrophil activation on TNF-stimulated endothelial cells. Using a flow-based adhesion assay, neutrophils isolated from whole blood were perfused across endothelial cells. Neutrophils were recruited to endothelial cells treated with TNF, but did not adhere to unstimulated endothelium. Detailed analysis of neutrophil behaviour showed that on TNF-stimulated endothelial cells, the majority of recruited cells transmigrated across the monolayer. When neutrophils were treated with anti-CXCR2, but not anti-CXCR1, neutrophil activation was inhibited so that nearly all of the recruited neutrophils rolled indefinitely on the monolayer and could not migrate; data are mean±SEM of four experiments **p<0.01 for comparison by paired *t*-test of neutrophil behaviour on TNF stimulated endothelial cells in the presence or absence of anti-CXCR2.

In vertebrates, tissue trauma or infection causes the rapid initiation of an inflammatory reaction. The early phase of this phylactic response results in the localised recruitment of cells of the innate immune system from the blood, a tissue infiltrate that is dominated by neutrophils. The molecular processes that support the initial interactions between blood-borne neutrophils and the endothelial cells lining postcapillary venules (the site in the blood vasculature where leukocytes are recruited during inflammation) are well described (see Figure 1 for a schematic representation of the steps in the neutrophil recruitment process). In response to the localised production of inflammatory mediators, such as the cytokine tumour necrosis factor-α (TNF), activated endothelial cells decorate themselves with specialised adhesion receptors of the selectin family [1]–[3]. Due to their ability to rapidly form strong but short-lived bonds with carbohydrate counter-ligands on the neutrophil surface, E- and P-selectin are capable of tethering neutrophils from rapidly flowing blood [4]. The sequential formation and dissolution of selectin bonds also support a characteristic and dynamic form of adhesion, referred to as *rolling* [5]. Rolling adhesion does not require neutrophil activation. However, neutrophil migration through the vessel wall and into the inflamed tissue is dependent upon the receipt of an activating stimulus [5],[6]. We have previously shown that endothelial cells can present peptide agonists of the CXC-chemokine family to rolling neutrophils and thus stabilise adhesion [7],[8]. Ligation of the neutrophil CXC-Receptor-2 (CXCR2) by these agents is essential for activation of the β2-intergrin adhesion receptors and the reorganisation of the actin cytoskeleton that support transendothelial cell migration [8]. Here, we show that in the presence of an antibody that blocks chemokine interactions with CXCR2, neutrophils roll on the endothelial cell surface indefinitely (Figure 2), demonstrating that a chemokine signal is essential for neutrophil activation on TNF-stimulated endothelial cells. However, the removal of this primary activating stimulus provides no information on the requirement for additional, downstream signals, which might coordinate transit of the vessel wall and onward migration into stromal tissues.

## Results/Discussion

### EPA Released from Endothelial Cell Membrane Phospholipids Inhibits the Recruitment of Neutrophils

**Figure 3:**
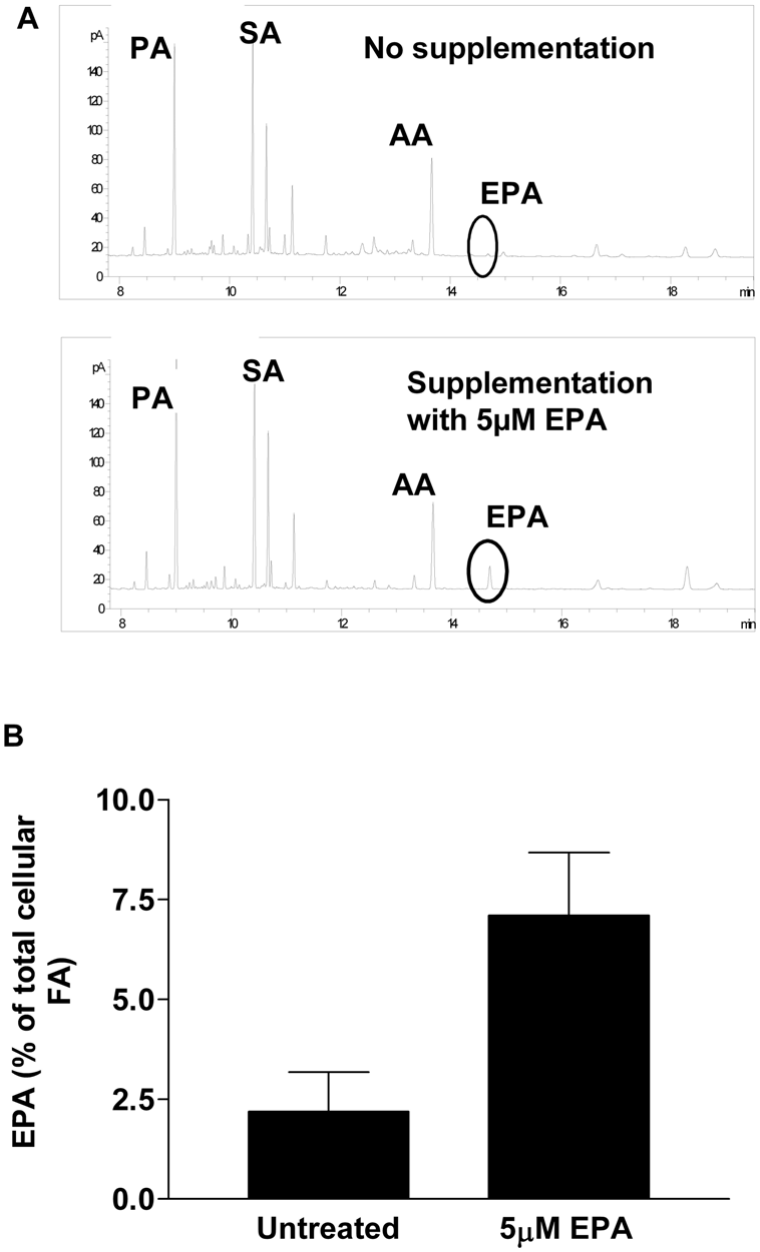
The effects of EPA supplementation on the fatty acid content of endothelial cell phospholoipds. (A and B) The concentration of EPA in membrane phospholipids was increased after supplementation of culture medium with EPA for 24 h. Error bars indicate SEM. AA, arachidonic acid; FA, fatty acid; PA, palmitic acid; SA, stearic acid.

**Figure 4:**
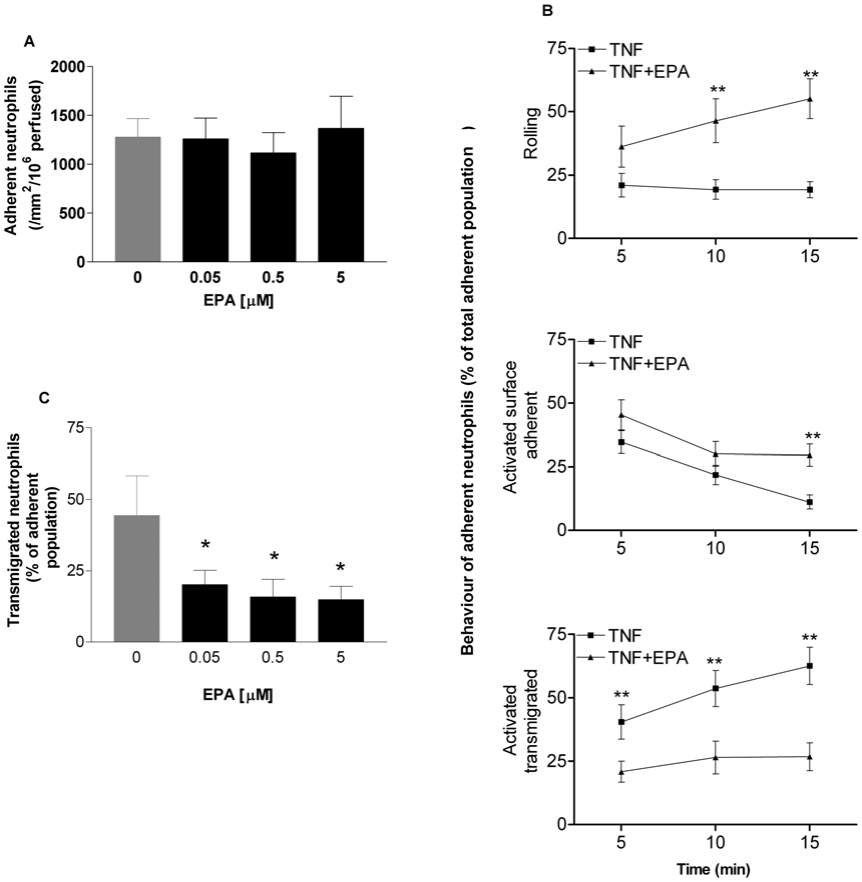
The effects of EPA supplementation on the adhesive behaviour of neutrophils. (A) EPA supplementation did not affect the number of neutrophils initially adhering to endothelial cells from flow. (B) However, the time course of neutrophil behaviour on TNF-stimulated endothelial cells showed that EPA supplementation drastically altered neutrophil behaviour. Thus, the number of activated and surface adherent neutrophils decreased with time in the presence or absence of EPA. However, on endothelium that had not been supplemented with EPA, this was because neutrophils transmigrated across the monolayer, whereas on EPA-supplemented endothelium this was because activated cells reverted back to a rolling form of adhesion. ANOVA showed that there was a significant effect of treatment (i.e., ±EPA) on the percentage of cells that were rolling, surface adherent, or transmigrated (*p*<0.01). In addition, there was a significant effect of time on each form of behaviour for the EPA-treated cells (*p*<0.05−0.01). Bonferroni tests showed significant differences at specific time points as marked; **p<0.01. (C) Inhibition of neutrophil transmigration was evident at levels of EPA supplementation as low as 50 nM. Data are mean±SEM of five experiments. ANOVA showed significant effects of treatment (*p*<0.05); *p<0.05 compared to untreated control by Dunnett's test. Error bars indicate SEM.

Dietary omega-3 polyunsaturated fatty acids (n-3-PUFAs) have anti-inflammatory properties. For example, their inclusion in the diet in the form of n-3-PUFA–rich fish oil reduces the symptoms of disease as well as the use of nonsteroidal anti-inflammatory drugs in arthritis patients with severe inflammatory joint disease [9]. In addition, dietary n-3-PUFAs may offer protection against vascular pathology associated with atherosclerosis, having been reported to be efficacious in epidemiological studies [10]. By modulating inflammation within the artery wall, n-3-PUFAS also alter the cellular and the structural composition of advanced atherosclerotic plaques in a manner that could reduce the incidence of plaque rupture or ulceration, a process that precedes tissue infarction (e.g., heart attack or stroke) [11]. Despite these documented anti-inflammatory benefits, the mode(s) of interaction of these lipids with the immune and inflammatory systems are not well understood. When we supplemented endothelial cell culture medium with the major n-3 -PUFA found in dietary fish oil supplements, eicosapentaenoic acid (EPA; 20:5n-3), it was incorporated into cellular phospholipids so that upon withdrawal of the free fatty acid from the culture medium, a pool of esterified EPA remained localised within endothelial cell membranes (Figure 3A and 3B). After withdrawal of free fatty acid and stimulation with TNF, EPA-treated cells were able to support similar levels of neutrophil adhesion to those that had received no lipid supplement (Figure 4A). However, detailed analysis showed that the behaviour of neutrophils on the two populations of endothelial cells was very different. In response to TNF, but in the absence of EPA, a small population of adherent neutrophils (∼20%) rolled throughout the duration of a flow adhesion assay (Figure 4B). The remaining cells became activated on the endothelial cell surface, and this population of neutrophils became progressively smaller as cells migrated across the endothelial cell monolayer (Figure 4B). In contrast, when endothelial cells had been treated with EPA prior to TNF stimulation, there was a marked reduction in the number of cells undergoing transendothelial cell migration (Figure 4B). This was reduced at the earliest time point and did not increase over the duration of the experiment. Interestingly however, many of the cells that did become activated on the surface of endothelial cells reverted to a rolling form of adhesion (Figure 4B), implying that after the receipt of an initial chemokine stimulus, a second signal was required to allow prolonged adhesion and migration across the endothelial cell monolayer. Experiments using different concentrations of EPA showed that these inhibitory effects were evident at concentrations as low as 50 nM (Figure 4C).

### EPA Does Not Inhibit the Expression of Inflammatory Genes in Endothelial Cells

**Figure 5:**
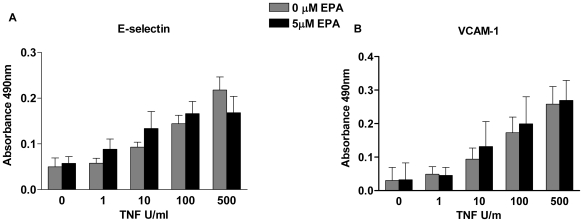
The effect of EPA on TNF-induced surface adhesion molecule expression. The expression of (A) E-selectin and (B) VCAM-1 was measured by ELISA in TNF-stimulated endothelial cells that were either unsupplemented or supplemented with EPA. Data are mean±SEM of seven experiments. ANOVA showed that there was a significant effect of the dose of TNF (*p*<0.01), but not of EPA, on the expression of both receptors.

To define the molecular mechanisms underlying these observations, we tested two prevalent hypotheses. The first, predicated on the observation that n-3-PUFAs regulate the transcription of endothelial cell inflammatory genes by down-regulating the activity of the nuclear factor-κB, predicts changes in the levels of adhesion receptor and chemokine expression after supplementation with EPA [12]–[14]. However, previous studies have investigated the regulation of gene expression in the presence of up to 100 µM of n-3-PUFAs, whereas physiological blood plasma levels of free fatty acid are of the order of 1 µM [15], a level that can be increased several fold upon supplementation. Using 5 µM EPA as an approximation of the highest concentration achievable in vivo, we did not see changes in the expression of endothelial cell adhesion molecules when assessed for RNA transcripts or protein expression (Figure 5 and Table 1). Neither was there any alteration in the levels of cytokines and chemokines secreted by endothelial cells (Table 2). Thus, in this model, we were able to discount the gene regulation hypothesis.

**Table 1:** Effect of EPA treatment on mRNA expression of endothelial cell adhesion molecules and chemokines induced by TNF.

Analyte	TNF-α	TNF-α+5 µM EPA	Statistics
E-selectin	2,046±780	1,890±854	NS
ICAM-1	55±13	61±21	NS
VCAM-1	52±16	64±26	NS
TNF-α	16±11	17±13	NS
MCP-1	5±2.	9±3	NS
CXCL1	1±0.05	1±0.04	NS
CXCL2	8±3	7±2	NS

Endothelial cells were cultured alone or in the presence of 5 µM EPA for 24 h and stimulated with 100 U/ml TNF for the final 4 h. Data are presented as the fold change compared to the level of mRNA in unstimulated endothelial cells. There was no significant effect of EPA treatment on the gene expression profile of TNF-stimulated endothelial cells. Data are ±SEM of four experiments.

NS, not significant.

**Table 2:** Effect of EPA treatment of endothelial cells on the TNF-α-induced secretion of cytokines and chemokines.

Analyte	TNF-α	TNF-α+0.5 µM EPA	TNF-α+5 µM EPA	Statistics
IL-6	204±19	156±8	180±23	NS
IL-8	5,695±52	5,302±561	5,384±891	NS
IFN-γ	3±0.5	2.0±0.2	2±0.2	NS
CXCL10	112±17	100±29	136±15	NS
GM-CSF	8±2	6±1	7±1	NS
CCL2	8,298±854	6,000±342	7,411±420	NS
CCL3	236±97	230±21	265±130	NS
CCL5	386±32	406±128	640±121	NS
TNF-α	358±78	225±36	252±31	NS
TNF-β	4±0.3	3±1	4±0.5	NS

Endothelial cells were cultured alone or in the presence of 5 µM EPA 24 h and stimulated with 100 U/ml TNF for the final 4 h. Culture supernatants were collected and assayed by Luminex for cytokines and chemokines. There was no significant effect of EPA treatment on analyte expression compared to TNF-stimulated endothelial cells. Data are presented as picograms per milliliter, and are mean±SEM of four experiments.

NS, not significant.

### The Anti-Inflammatory Effects of EPA Supplementation Require the Metabolic Activity of Cyclooxygenase Enzymes

**Figure 6:**
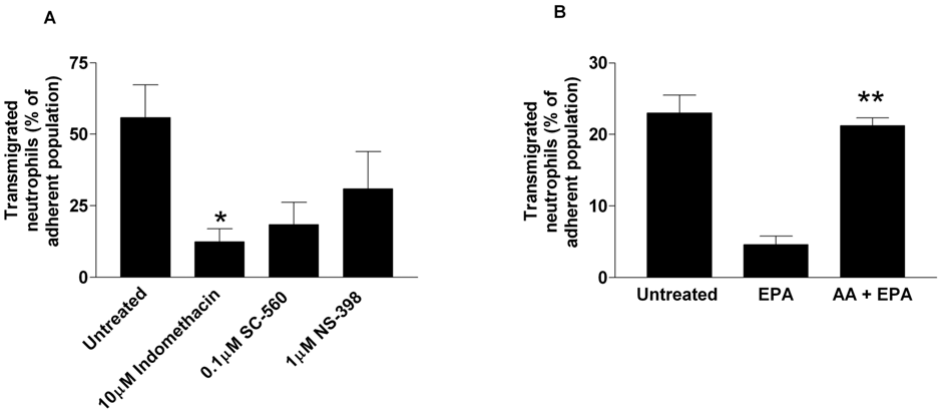
A cyclooxygenase (COX)-derived eicosanoid is required for neutrophil transmigration across TNF-stimulated endothelial cells. (A) Inhibition of COX-1 (SC-560), COX-2 (NS-398), or both (indomethacin) caused inhibition of neutrophil transmigration, *p<0.05 by *t*-test. (B) Addition of AA to EPA-supplemented endothelial cells during the period of TNF stimulation restored neutrophil transmigration. All data are mean±SEM of four experiments. ANOVA showed significant effects of treatments in both (A and B) (*p*<0.01). In (A), Dunnett test showed significant effects of individual treatments compared to untreated control, *p<0.05. In (B), Dunnett's test showed significant difference between EPA-treated and untreated control (**p<0.01), but not between AA+EPA and control.

The second hypothesis proposes that upon endothelial cell activation, EPA may compete with the n-6-PUFA arachidonic acid (AA; 20:4n-6) for cyclooxygenase enzymes (COX1 and COX2) after both fatty acids are liberated from membrane phospholipids by endogenous phospholipases [16]. Ordinarily, AA is metabolised by COX into 2-series endoperoxides, with downstream synthases converting these into the biologically active 2-series prostanoids [17]. However, when utilised as a COX substrate, the metabolism of EPA generates alternative 3-series prostanoids [16]. The 2-series prostanoids generated by COX activity are known to regulate aspects of the inflammatory response. For example, in the COX2 knockout mouse, neutrophil recruitment is dramatically reduced upon ischaemia and reperfusion-induced injury of the liver when compared to wild-type control animals [18]. In a murine model of lipopolysaccharide-induced lung inflammation, inhibitors of COX function (indomethacin and aspirin) modestly increased neutrophil recruitment [19]. Conversely in vitro, inhibition of COX function by aspirin can inhibit neutrophil migration [20] while having no effect on the levels of neutrophil adhesion to the endothelial cell monolayer [21]. Moreover, in murine models of acute and chronic inflammation, a reduction in PGE_2_ production by genetic deletion of its membrane-bound synthase, moderates the formation of inflammation-associated granulation tissue and angiogenesis, as well as decreasing the nociception of pain, indicating that PGE_2_ is proinflammatory in these models [22]. PGD_2_ also appears to have proinflammatory functions, as overexpression of the synthase generating this prostanoid increases production of inflammatory cytokines and chemokines, leading to exaggerated levels of eosinophil and lymphocyte recruitment [23], a mechanism that operates through the PGD_2_ receptor DP-2. In contrast, in acute peritoneal inflammation, knockout of PGD_2_ synthase increased inflammatory cytokine production and retarded the rate of inflammatory resolution by a mechanism that operated through the DP-1 receptor [24]. Thus, PGD_2_ appears to have pro- or anti-inflammatory capabilities depending on the nature of the inflammatory insult. The inflammatory activity of the equivalent 3-series prostanoids is not known. Here, by introducing a panel of COX inhibitors into endothelial cell cultures at the same time as they were activated with TNF, the effects of EPA supplementation could be replicated, with neutrophil transmigration being dramatically inhibited (Figure 6). Moreover, adding a molecular excess of AA to EPA-supplemented endothelial cells at the point of TNF activation could reverse the blockade of migration (Figure 6). Taken together, these data imply that a COX-derived product of AA is required for the transmigration of neutrophils across TNF-stimulated endothelial cells, and that in the presence of EPA, this pathway is efficiently antagonised.

### Prostaglandin D2 Is Required for Neutrophil Migration across Endothelial Cells Stimulated with TNF-α

Endothelial cells generate COX products constitutively. For example, prostacyclin (PGI_2_) and prostaglandin D_2_ (PGD_2_) are endothelial cell-derived vasoactive prostanoids that are also involved in the regulation of haemostasis, being antagonists of platelet activation [17],[25]. These prostanoids are difficult to measure in the systemic blood, as they have reported half-lives in plasma that are measured in minutes [26],[27]. However, the constitutive nature of their production is demonstrated by the presence in urine and body fluids of their downstream metabolic products, PGF_1α_ and delta-PGJ_2_, respectively [28],[29].

Prostanoids have a documented ability to regulate the migration of a number of leukocyte subsets. For example, PGD_2_ induces chemotaxis of eosinophils and T-lymphocytes [30] in vitro. Interestingly, the migration of monocyte-derived migratory dendritic cells (DCs) may be tightly regulated by interplay between different prostanoids. Thus, the ability of these cells to traffic out of tissue into lymph nodes via the lymphatic circulation is dependent upon the presence of PGE_2_ as a differentiation signal [31]–[34]. In this context PGE_2_ promotes the function (but not the expression) of the chemokine receptor CCR7, so that DCs efficiently respond to the chemokines CCL19 and CCL21. Importantly, a number of studies have shown that the presence of PGD_2_ can in turn inhibit the ability of DCs to migrate out of the lungs or the skin during an inflammatory response, although the molecular mechanism by which this inhibition is achieved remains undescribed [35],[36].

**Figure 7:**
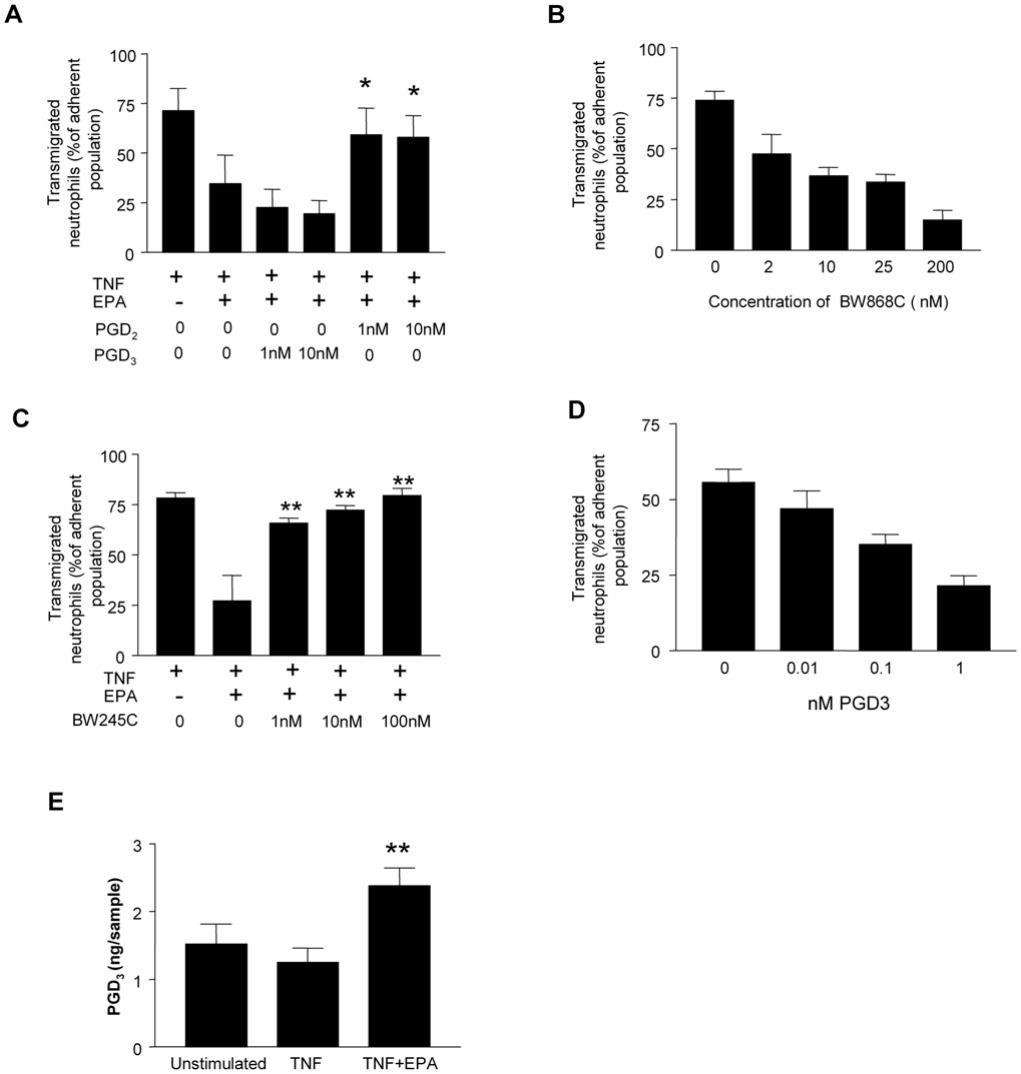
The role of AA- and EPA-derived eicosanoids in neutrophil transmigration. (A) When PDG_2_ was perfused across a population of neutrophils adherent to EPA-treated endothelial cells, neutrophil transmigration was restored. However, PGD_3_ had no significant effect on neutrophil behaviour. All data are mean±SEM of four experiments. ANOVA showed significant effect of treatment on transmigration. *p<0.05 compared to EPA-supplemented endothelial cells in the absence of PGD_2_ by Dunnett test. (B) The DP1 receptor antagonist BW868c dose-dependently inhibited neutrophil transmigration on TNF-stimulated endothelial cells. All data are mean±SEM of five experiments; ANOVA showed a significant effect of BW868c concentration (*p*<0.01). (C) Perfusion of the DP-1 receptor agonist, BW245C, across neutrophils adherent to EPA-supplemented endothelial cells restored neutrophil migration to control levels. Data are mean±SEM of five experiments. ANOVA showed a significant effect of treatment on transmigration. **p<0.01 compared to EPA-supplemented endothelial cells in the absence of BW868C by Dunnett test. (D) Neutrophils perfused across TNF-stimulated endothelial cells in the presence of PGD_3_ showed a significantly reduced ability to migrate across the monolayer. Data are mean±SEM of three experiments; ANOVA showed a significant effect of PGD_3_ concentration on transmigration (*p*<0.01). (E) The levels of PGD_3_ released from endothelial cells are increased after EPA supplementation. Data are mean±SEM of three experiments. ANOVA showed a significant effect of treatment (*p*<0.05). **p<0.01 for PGD3 production compared to endothelial cells activated with TNF without EPA supplementation by Dunnett's test.

**Figure 8:**
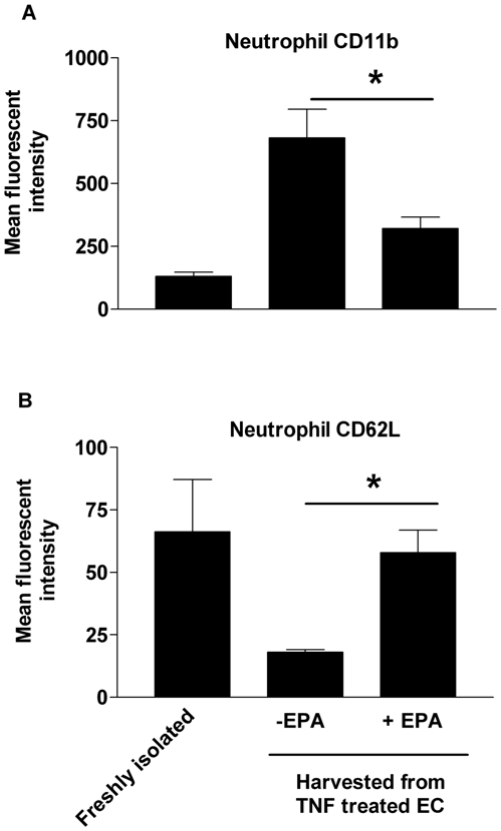
The effect of adhesion on the expression of l-selectin and CD11b on neutrophils. Neutrophils that were freshly isolated, migrated across endothelial cells stimulated with TNF or adherent to the surface of EPA-supplemented endothelial cells (EC) activated with TNF were harvested and the expression of (A) CD11b or (B) l-selectin (CD62L) assessed by flow cytometry. Data are mean±SEM of four experiments; ANOVA showed significant effect of treatments in (A) (*p*<0.01) and (B) (*p*<0.05). In (A and B), Dunnett's test showed significant difference between −EPA and freshly isolated cells (*p<0.01 and *p<0.05, respectively), but not between +EPA and freshly isolated cells.

As prostanoids are well documented to regulate leukocyte migration [37], we tested the hypothesis that PGD_2_ was the endothelial cell-derived agent providing the stimulus for neutrophil migration across the monolayer. We established a population of surface adherent neutrophils on TNF-treated endothelial cells supplemented with EPA and perfused synthetic PGD_2_ across the endothelial cells and neutrophils to see whether this would reintroduce transmigration. The provision of exogenous PGD_2_ but not PGD_3_ (which could be derived from EPA), fully restored the ability of neutrophils to cross the endothelial cell monolayer (Figure 7A). Prostaglandin D_2_ has two receptors. Chemoattractant-receptor homologous molecule expressed on Th2 cells receptor (CRTH2 or DP-2) is not expressed in neutrophils [35], whereas the DP-1 receptor has been reported in these cells [38]. Here, neutrophils perfused across TNF-stimulated endothelial cells in the presence of a DP-1 receptor antagonist (BW868C) showed a greatly diminished efficiency of endothelial cell transmigration (Figure 7B). Conversely, the transmigration of neutrophils was re-established on EPA-supplemented endothelial cells when a synthetic DP-1 receptor agonist (BW245C) was perfused across cells adherent to the surface of the monolayer (Figure 7C). Importantly, we could demonstrate that PGD_2_ was operating directly on the neutrophils, because when these cells were harvested after migrating across a TNF-stimulated endothelial cells they had up-regulated CD11b (the alpha subunit of the β2 integrin CD11b/CD18 that is required for efficient neutrophil transmigration) and proteolytically shed l-selectin (Figure 8). However, neutrophils harvested from the surface of EPA-treated endothelial cells did not shed l-selectin or up-regulate CD11b. Thus, in this assay system, the delivery of a PGD_2_-mediated signal to the neutrophils was necessary for full cellular activation and efficient transendothelial migration.

### PGD_3_ Generated from EPA Antagonises the PGD_2_-Mediated Migration of Neutrophils across Endothelial Cells

Taken together, these data show that PGD_2_ operates through the neutrophil DP-1 receptor to provide a signal that is essential for transendothelial cell migration. Moreover, the ability of neutrophils to respond to this signal is abolished when endothelial cells are preloaded with EPA from which PGD_3_ is generated. This implies that PGD_3_ may be an effective antagonist of the DP-1 receptor. To test this hypothesis, we perfused neutrophils across TNF-stimulated endothelial cells in the presence of exogenous PGD_3_. Neutrophil transmigration was inhibited in a dose-dependent manner by PGD_3_, showing that in the presence of endogenously generated PGD_2_ it could effectively antagonise this process (Figure 7D). Importantly, we were able to show that cells supplemented with EPA-generated an increased amount of PGD_3_ (Figure 7E).

The absolute levels of PGD_2_ and PGD_3_ that are generated in our assay system, and thus the ratio of their abundance, are not easily assessed. The levels of PGD_2_ measured as an endothelial cell releasate were at the limits of detection (Table 3), presumably due to the short half-life of this prostanoid under physiological conditions; however, significant levels of PGD_3_ were assayed upon EPA supplementation, and concentrations in the order of 5–10 nM had accumulated over the 24 h of the EPA supplementation period. The half-life of PGD_3_ in serum or albumin-containing medium has not been reported to our knowledge; thus it is not clear whether the measurements made here report the true levels of PGD_3_ production, or if metabolic degradation of this prostanoid renders these measurements underestimates. Irrespective of this, our experiments utilising synthetic PGD_2_ demonstrate that concentrations on the order of 1 nM were sufficient to fully recapitulate the process of migration on EPA-supplemented endothelial cells, and when using PGD_3_ as an antagonist of TNF-induced transmigration marked effects were evident at 10–100 pM. Thus, the measurable levels of PGD_3_ that we report would certainly be sufficient to effectively antagonise the transmigration of neutrophils in our experimental model.

**Table 3:** Analysis of eicosanoids by liquid chromatography tandem mass spectrometry.

Treatment	5-HETE	8-HETE	11-HETE	12-HETE	15-HETE	PGD_2_/PGE_2_
Untreated	3.8±1.5	0.5±0.2	1.0±0.4	2.7±1.0	1.4±0.5	0.074±0.12
100 U/ml TNF-α	2.6±0.6	0.4±0.1	0.7±0.1	2.0±0.4	1.0±0.2	0.066±0.01
100 U/ml TNF-α+5 µM EPA	2.3±0.6	0.3±0.1	0.7±0.2	1.9±0.5	1.0±0.2	0.064±0.01

HETEs and prostanoids were analysed by reverse-phase liquid chromatography tandem mass spectrometry in unstimulated and TNF-stimulated endothelial cells in the presence or absence of EPA. There was no significant difference in concentrations of HETEs or prostanoid secretion under any treatment (statistical analysis by ANOVA). Data are presented as nanograms per sample and are mean±SEM of three experiments.

### Conclusions

Our study demonstrates a hitherto unknown regulatory step in the recruitment of neutrophils by cytokine-stimulated endothelial cells (refer to Figure 1 to see how this new step fits with the known regulatory mechanisms of neutrophil recruitment). After initial tethering by selectin molecules, a chemokine signal induces arrest of the cell on the endothelial cell surface. However, the chemokine signal is not sufficient to support transmigration across the endothelial cell monolayer, and the arachidonic acid-derived prostanoid, PGD_2_, is an essential downstream regulator of this process. PGD_2_ operates through the DP-1 receptor and this signal can be effectively antagonised by PGD_3_ which is generated from EPA released by the action of phospholipase enzymes on the phospholipids of EPA supplemented cells. Not only does this study reveal a new step in the recruitment of neutrophil recruitment during inflammation, it also reveals a novel anti-inflammatory mechanism of action of the dietary n-3-PUFA, EPA.

## Materials and Methods

### Cell Culture and Neutrophil Isolation

Endothelial cells (EC) were isolated and cultured, as previously described [39], with or without 0–5 µM EPA (Sigma) for 24 h. TNF-α (100 U/ml; R&D systems) was added to the EC for the final 4 h of culture. In some experiments, EC were treated with 100 U/ml TNF-α in the presence of 10 µM indomethacin or 0.1 µM SC-560 or 1 µM NS-398 (Sigma). In the AA reconstitution experiments, 5 µM AA (Sigma) was added to the culture medium simultaneously with TNF-α.

Blood from healthy adult volunteers was collected into tubes coated with EDTA (1.6 mg/ml), and neutrophils were separated using 2-step density gradients of Histopaque 1119 and 1077 (Sigma), as previously described [40]. After washing in 0.15% BSA in PBS, cells were counted using a Coulter Multisizer (Coulter Electronics), and neutrophils, which were >95% pure, were resuspended at a concentration of 1×10^6^/ml in PBS/Alb with calcium and magnesium.

### RNA Extraction and Real-Time PCR

mRNA was isolated from EC using the Qiagen RNEasy Mini Kit 50 (Qiagen) following the manufacturer's instructions. Real-time PCR (RT-PCR) was performed using QuantiTect probe RT-PCR kit according to the manufacturer's instructions (Qiagen). Primers were purchased from Applied Biosystems. The expression of each target gene was normalised to β-actin expression, and the data presented represent fold change compared to untreated EC.

### Luminex for Secreted Cytokines and Chemokines

Supernatants were collected from EC that had been incubated with 0–5 µM EPA for 24 h and had 100 U/ml TNF added to the culture for the final 4 h of culture. The Luminex kit was purchased from Upstate/Chemicon, and the experiment performed to the manufacturer's instructions. Data were collected and analysed from the samples using a Luminex100 machine (Luminex).

### Flow-Based Adhesion Assay

Glass capillaries (microslides) containing treated EC monolayers were incorporated into a flow-based adhesion assay as described [39]. Briefly, microslides were attached to cell and fluid reservoirs by silicon tubing at one end and to a withdrawal syringe pump at the other end. After mounting on the stage of a phase contrast microscope, EC were washed for 2 min with PBS containing 0.15% BSA. Neutrophils were then perfused (1×10^6^/ml) at 0.1 Pa for 4 min, followed by 15 min of wash. Video images were recorded throughout the experiment and neutrophil behaviour analysed offline using Image Pro software (Image-Pro Plus). In some experiments, neutrophils were perfused across EPA- and TNF-treated EC, allowed to adhere, and subsequently perfused with 1 nM PGD_2_ or 1 nM PGD_3_ (both from Cayman Chemicals). Alternatively, neutrophils were treated with BW868c (Cayman Chemicals), a DP1 receptor antagonist, for 10 min prior to perfusion over TNF-α-stimulated EC. Neutrophils were also treated with the DP1 receptor agonist BW245c (Cayman Chemicals) prior to perfusion over EPA- and TNF-α-treated EC. Neutrophils were also treated with 0–1 nM PGD_3_, immediately prior to perfusion across TNF-stimulated EC. In all experiments, lipid reagents, agonists, and antagonists were stored in 100% ethanol under nitrogen gas. Once diluted, the medium contained <0.01% ethanol. All controls contained equivalent concentrations of ethanol.

### Analysis of EPA Incorporation into Cellular Phospholipids

First-passage confluent EC were treated with 5 µM of EPA for 24 h. EC were removed from the plastic culture dish by scraping and stored in 0.88% KCl solution at −20°C until analysis. Total lipid was extracted with chloroform∶methanol (2∶1, v/v) containing butylated hydroxytoluene (50 mg/l) as antioxidant. Fatty acids were subsequently hydrolysed from the lipid and simultaneously methylated by incubation with methylation reagent (methanol containing 2% v/v H_2_SO_4_) at 50°C for 2 h. Fatty acid methyl esters were separated and identified using a Hewlett Packard 6890 gas chromatograph (Hewlett Packard) fitted with a 30 mm×32 mm BPX 70 capillary column, film thickness 0.25 µm. Helium, at the initial flow rate of 1.0 ml/min, was used as the carrier gas. Injector and detector temperatures were 275°C, and the column oven temperature was maintained at 170°C for 12 min after sample injection. The oven temperature was programmed to increase from 170 to 210°C at 5°C/min. Fatty acid methyl esters were identified by comparison with authentic standards. Peak areas were quantified using ChemStation software (Hewlett Packard). Each fatty acid was expressed as weight percent of total fatty acids present.

### Eicosanoid Extraction and Reverse-Phase High-Performance Liquid Chromatography Analysis

Eicosanoids were extracted from control EC using C18 Sep-Pak cartridges (Waters). Briefly, cartridges were conditioned with 5 ml of high-performance liquid chromatography (HPLC)-grade MeOH and rinsed twice with water. Reactions were terminated with addition of 100% ice-cold MeOH, containing 2 ng of internal standard PGE2-d4. Sample volume was adjusted to 10% MeOH with water and applied to the column. The column was rinsed with water and the sample eluted with 2 ml of MeOH. Nitrogen gas was used to dry the sample, which was resuspended in 100 µl of MeOH and stored at −80°C until analysis.

### Prostaglandin Quantitation using Liquid Chromatography Tandem Mass Spectrometry

Prostaglandins were separated on a C18 ODS2, 5 µm, 150×4.6-mm column (Waters) using a gradient of 50%–90% B over 20 min (A, water∶acetonitrile∶acetic acid, 75∶25∶0.1; B, methanol∶acetonitrile∶acetic acid, 60∶40∶0.1) at 1 ml/min. Products were quantitated by directing the HPLC output directly into the electrospray source of a Q-Trap mass spectrometer (Applied Biosystems 4000 Q-Trap) operating in the negative mode (−4,500 V). Individual prostaglandins were monitored in the Multiple Reaction Monitoring (MRM) mode using specific parent to daughter transitions of *m*/*z* 349–269 for PGD_3_ with collision energies of −26 V. Products were identified and quantified with reference to the appropriate standards run in parallel under the same conditions, with 2 ng of PGE2-d4 (*m/z* 355–275) added as an internal standard.

### Analysis of Neutrophil Adhesion Molecule Expression by Flow Cytometry

Neutrophils for analysis were isolated from whole blood as described above. Control cells were stained for flow cytometry directly after isolation. Cells rolling on EPA-treated endothelial cells in microslides were harvested by elution with 0.02% EDTA after 4 min of neutrophil perfusion and 11 min of wash buffer perfusion (totalling 15 min of contact with endothelial cells). In order to harvest neutrophils that had migrated across endothelial cells, endothelium was cultured on 1.5 mg/ml type I collagen gels (Becton Dickinson). After endothelial cell stimulation with TNF for 4 h, neutrophils were added for 15 min. Surface-adherent cells were washed off with 0.02% EDTA, and the gel was dissolved using type VII collagenase (Sigma). Neutrophils were harvested from the dissolved gel by centrifugation. All neutrophil populations were stained for surface expression of CD11b (PE-conjugated, clone 2LPM19c, 2 µg/ml; DAKO) or CD62L (FITC-conjugated, clone Dreg56,10 µg/ml, Beckton Dickinson). Expression was analysed by flow cytometry using a DAKO CyAn, and data analysed using Summit software (Becton Dickinson). Data are expressed as mean fluorescent intensity.

### Statistical Analysis

Data were analysed using Prism software (GraphPad software). Results are presented as means±standard error of the mean (SEM). Comparisons between individual treatments were by paired *t*-test where appropriate. ANOVA was performed to assess the effect of EPA concentration on PMN transmigration. Significant findings were investigated further using Bonferroni multiple comparison test or Dunnett's test.

## Abbreviations

DC: dendritic cell
